# Identifying postnatal anxiety: comparison of self-identified and self-reported anxiety using the Edinburgh Postnatal Depression Scale

**DOI:** 10.1186/s12884-022-04437-0

**Published:** 2022-03-03

**Authors:** Gracia Fellmeth, Siân Harrison, Jenny McNeill, Fiona Lynn, Maggie Redshaw, Fiona Alderdice

**Affiliations:** 1 National Perinatal Epidemiology Unit, Nuffield Department of Population Health, University of Oxford, Richard Doll Building, Old Road Campus, Oxford, OX3 7LF UK; 2 School of Nursing and Midwifery, Queens University Belfast, Belfast, Northern Ireland UK

**Keywords:** Anxiety, Perinatal, Edinburgh postnatal depression scale (EPDS), Self-reported, Self-identified, Screening

## Abstract

**Background:**

Identifying women with perinatal anxiety is important in order to provide timely support and prevent adverse outcomes. Self-report instruments are commonly used in maternity settings. An alternative is to ask women directly whether they self-identify as having anxiety. We examine the agreement between self-reported and self-identified anxiety at 3 months postpartum and compare the characteristics of women with self-reported and self-identified anxiety.

**Methods:**

A secondary analysis of national maternity surveys conducted in 2014 in England and Northern Ireland was conducted. Self-reported anxiety was assessed using the Edinburgh Postnatal Depression Scale anxiety subscale (EPDS-3A). Agreement between self-reported and self-identified anxiety was measured using Cohen’s kappa. Logistic regression was used to identify characteristics of women in each group.

**Results:**

In our sample of 6752 women, 14.2% had self-reported anxiety, 5.9% had self-identified anxiety and 3.5% were positive on both measures. Among those with self-identified anxiety, 58.1% also had self-reported anxiety. Of those with self-reported anxiety, 24.4% also had self-identified anxiety. Statistical agreement between the two measures was minimal with Cohen’s kappa 0.283 at an EPDS-3A threshold of ≥6. Among both self-identified and self-reported anxiety groups, psychological factors were the strongest associated factors. Women with self-reported anxiety had higher odds of being from Northern Ireland (OR 1.81); having a mixed or unhappy reaction to the pregnancy (OR 1.65); living without a partner (aOR 1.37); and antenatal depression (aOR 1.32). Women with self-identified anxiety had higher odds of physical problems (OR 1.84); and being of Black or minority ethnicity (OR 0.39).

**Conclusions:**

Asking postnatal women directly whether they self-identify as having anxiety identifies a different group of women from those who score highly on self-report measures. Women with self-identified anxiety may benefit from further follow-up and support.

## Background

The perinatal period is a time of risk for the development, recurrence and exacerbation of anxiety, depression and other common mental disorders. Anxiety disorders are typically characterised by symptoms of physiological arousal, cognitive distortions and behavioural avoidance [1]. Although anxiety is more prevalent during the perinatal period, there is no evidence to suggest that the qualitative experience of anxiety during the perinatal period differs from anxiety at other stages of life. Anxiety in pregnancy and the postnatal period represents a significant burden among women: estimates suggest prevalence between 18 and 25% in pregnancy and 15% postnatally [2, 3]. These estimates are based on meta-analyses which include heterogeneous studies with diverse populations and different assessment methods. Evidence suggests “true” prevalence varies broadly across samples and pooled estimates from meta-analyses mask significant variation between settings [3]. Early identification of women with anxiety is important in order to minimise suffering and distress for women and prevent adverse outcomes for their infants. However, the diagnosis of perinatal anxiety is often challenging [1]. The gold standard for identifying anxiety is through a clinical interview administered by a trained health professional to diagnose the presence of an anxiety disorder [4]. However, clinical interviews are time-consuming and resource-intense and as such not always a feasible or appropriate means of identifying women with anxiety, especially in non-specialist services such as maternity care. Alternative approaches are therefore required to identify women with anxiety in maternity settings.

The most commonly used method of identifying women with anxiety in maternity care settings is self-report instruments. These instruments screen for the presence of anxiety symptoms. They have validated cut-off points: scores above this threshold may indicate the presence of an anxiety disorder and anyone scoring above the cut-off requires further investigation in the form of a clinical interview to confirm a diagnosis of anxiety disorder. This is the approach adopted in the United Kingdom, for example, where the National Institute for Health and Care Excellence (NICE) recommends all pregnant women are asked case-finding questions for anxiety and those who answer ‘yes’ to both questions are referred for a clinical interview [5, 6]. However, access to specialist perinatal mental health teams is not consistent across the UK and in some settings, further investigation of women who score above the threshold may be limited due to a lack of resources or insufficient maternal mental health service capacity. In these contexts, self-report measures are sometimes used in isolation with women scoring above the threshold considered to have ‘anxiety’ without any further investigation. This is problematic as it overlooks the importance of clinical judgement and details around the onset, course and duration of anxiety symptoms as well as the extent to which symptoms impact upon daily life [1]. Evidence shows that anxiety symptoms and scores and the extent of distress changes over time, with differences seen between antenatal and postnatal periods and particularly around the time of birth. Self-report measures provide only a snapshot in time; unless they are administered repeatedly, they may capture an atypical timepoint or transient symptoms. While this limitation also applies to other methods of case-finding, self-report measures are particularly prone as a result of their reliance on a rigid timeframe and cut-off value. Finally, there is cultural variation in the experience of anxiety, and self-report measures may be inadequate for capturing anxiety symptoms when used in contexts or populations that differ from the setting in which the instruments were developed [7, 8].

Another alternative to identifying women with anxiety is through a direct question asking women whether they self-identify as having anxiety. This offers a different insight from self-report instruments. The direct question elicits women’s own views with an emphasis on the subjective experience of anxiety as a whole rather than on the symptoms which constitute the disorder. Little is known about the utility and reliability of self-identified anxiety. In order to explore this, we use data from the National Maternity Surveys (NMS) in England and Northern Ireland to directly compare self-identified anxiety and self-reported anxiety among postnatal women. A previous analysis of the survey data compared self-identified and self-reported depression and found that, although they were related, statistical agreement between the two measures was minimal [9]. The current study aims to (i) determine the prevalence of self-identified and self-reported anxiety; (ii) establish the extent of agreement between these two measures; and (iii) compare the socio-demographic, clinical and psychological characteristics of women with self-identified anxiety and those with self-reported anxiety.

## Methods

### Study setting and participants

**Fig. 1:**
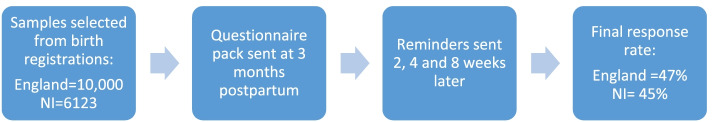
Survey methodology

This is a secondary analysis of two cross-sectional datasets from the United Kingdom: England’s *National Survey of Women’s Experience of Maternity Care 2014* and the *Northern Ireland Survey of Women’s Experience of Maternity Care 2014*. These surveys and datasets have been described in detail previously [10, 11]. Women were identified through birth registrations in England and Northern Ireland (Fig. 1). In England a random sample of 10,000 women who gave birth during a two-week period in January 2014 was selected. In Northern Ireland all women who gave birth between October and December 2014 were selected (*n* = 6123). Questionnaires were sent to women 3 months after they had given birth. Questions covered clinical events and care during pregnancy, labour, birth and the postnatal period and included self-identified physical and mental health outcomes. Women whose babies had died and those aged under 16 years were excluded. Reminders and additional questionnaires were sent two, four and eight weeks after the original mailing [12]. The questionnaire was also available online. Women did not receive any payment or incentive for completing the questionnaire. Usable response rates (excluding undeliverable questionnaires) were 47% (*n* = 4571) in England and 45% (*n* = 2722) in Northern Ireland [10, 11]. Across both surveys, respondents were more likely to be older, married and living in the least deprived areas when compared with non-respondents [10, 11]. This response pattern is in keeping with previous surveys [13–15].

### Assessments

The survey assessed both self-identified anxiety and self-reported anxiety. Self-identified anxiety was assessed using a single, direct question worded as follows: ‘Did you experience anxiety [10 days / 1 month / 3 months] after the birth of your baby?’. As the survey was administered at 3 months postpartum, the questions on anxiety at 10 days and 1 month postpartum relied on women’s recall. Responses were coded separately for each of the three time points. In addition, two composite variables were created: women who answered ‘yes’ at least once (either at 10 days, 1 month or 3 months post-partum) were classified as having *any* self-identified anxiety, and women who answered ‘yes’ at all three time points were classified as having *persistent* self-identified anxiety.

Self-reported anxiety was assessed using the Edinburgh Postnatal Depression Scale (EPDS) anxiety subscale. The EPDS is an established and validated tool for the identification of depression and anxiety symptoms in perinatal women [16]. The tool consists of ten items each scored 0–3; higher scores indicate a greater severity of symptoms. Three items on the EPDS are known as the anxiety subscale (EPDS-3A) because of the loading of these factors with anxiety symptoms [17–20]. These items are: “I have blamed myself unnecessarily when things went wrong” (item 3); “I have been anxious or worried for no good reason” (item 4); and “I have felt scared or panicky for no very good reason” (item 5) [16]. In the survey, women were asked to complete the full (ten item) version of the EPDS. However the current analysis was limited to responses to the three anxiety subscale items. In previous work, a threshold of ≥6 on the anxiety subscale has been found to maximise sensitivity and specificity for anxiety disorder among a sample of postpartum women in Australia [21]. This threshold was selected for the main analyses in the current paper. We also assessed the effect of applying thresholds of ≥5 and ≥ 7 in exploratory analyses.

The presence of the physical symptoms including painful stitches, incontinence and painful sexual intercourse was assessed by asking women if they experienced these at 10 days, 1 month or 3 months postpartum. Women were also asked whether they experienced depression, fatigue, sleep problems or post-traumatic stress disorder (PTSD)-type symptoms. PTSD-type symptoms were defined as experiencing any two of the following concurrently at 10 days, 1 month or 3 months postpartum: flashbacks, relationship problems and difficulty concentrating. Finally, women were asked whether they had experienced or sought help from a midwife or doctor for anxiety or depression during pregnancy. Those who answered ‘yes’ to experiencing or seeking help for either condition were defined as having experienced antenatal depression and antenatal anxiety [22].

### Statistical analysis

Datasets were cleaned and prepared individually before merging. The proportion of women with self-identified anxiety at 10 days, 1 month and 3 months postpartum were calculated. The proportion of women with self-reported anxiety was defined as those with EPDS-3A scores ≥6 at 3 months postpartum. We also assessed the proportion of women with EPDS-3A scores ≥5 and ≥7 to explore the impact of applying a lower or higher threshold. The extent of agreement between self-identified anxiety and self-reported anxiety at 3 months post-partum was explored by calculating the proportion of women with self-identified anxiety who also had self-reported anxiety and, conversely, the proportion of those with self-reported anxiety who also had self-identified anxiety. Self-identified anxiety at 3 months postpartum was selected for comparison because this assessed anxiety at the time of survey administration and provided a direct comparison with the self-reported EPDS-3A scores. Agreement between the two measures was quantified using Cohen’s kappa coefficient which takes into account the possibility of agreement occurring by chance [23]. Kappa coefficients were rated as: no agreement (0–0.20); minimal (0.21–0.39); weak (0.40–0.59); moderate (0.60–0.79); strong (0.80–0.90); and almost perfect (> 0.90) [23]. Logistic regression was used to identify demographic, clinical and psychological characteristics of women with self-identified anxiety and those with self-reported anxiety. Variables statistically significantly associated with the outcome in univariable analyses at *p* < 0.10 were retained for inclusion in the multivariable model. Variables were tested for collinearity using the test for pairwise correlation; when pairs of variables were strongly (correlation coefficient ≥ 0.8) and significantly (*p* < 0.05) correlated, only one was retained for the multivariable model. Full-case analysis was used. All analyses were conducted using STATA version 15.

## Results

Baseline characteristics of the sample of 7300 participating women have been published previously [9]. Over half (56.5%) of participants were aged 30–39 years, most (85.9%) had attended full-time education until over 17 years of age, 20.2% were born outside of the UK, 10.9% were of minority ethnic background and 49.1% were primiparous. EPDS data and the self-identified anxiety item were complete for 6752 women (92.5%); all subsequent analyses are limited to these complete cases.

### Prevalence of self-identified and self-reported anxiety

Table 1 summarises the prevalence of self-identified anxiety and self-reported anxiety. At 3 months post-partum, 5.9% (401/6752) of women had self-identified anxiety, 14.2% (957/6752) had self-reported anxiety and 3.5% (233/6752) were positive on both methods. Women with self-identified anxiety at 3 months had mean EPDS-3A scores of 5.7 (median 6, interquartile range 5–7) compared with mean scores of 2.7 (median 2, interquartile range 1–4; *p* < 0.01) among women without self-identified anxiety.

**Table 1:** Proportion and mean EPDS anxiety subscale (EPDS-3A) scores of women with self-identified and self-reported anxiety (*N* = 6752)

Definition	Proportion % (n)	Mean EPDS-3A score Mean (median; IQR)
**Self-identified anxiety**		
Anxiety at 10 days postpartum^a^	19.8 (1334)	4.5 (5; 3–6)
Anxiety at 1 month postpartum^a^	11.9 (803)	5.0 (5; 4–6)
Anxiety at 3 months postpartum^a^	5.9 (401)	5.7 (6; 5–7)
Any anxiety^b^	25.1 (1693)	4.6 (5; 3–6)
Persistent anxiety^c^	3.8 (255)	5.9 (6; 5–7)
**Self-reported anxiety**		
EPDS-3A score ≥5	24.2 (1637)	5.9 (6; 5–6)
EPDS-3A score ≥6	14.2 (957)	6.6 (6; 6–7)
EPDS-3A score ≥7	5.5 (372)	7.6 (7; 7–8)
**Both**		
Self-identified and self-reported anxiety (EPDS-3A ≥5)	4.5 (303)	6.6 (6; 6–7.5)
Self-identified and self-reported anxiety (EPDS-3A ≥6)	3.5 (233)	7.1 (7; 6–8)
Self-identified and self-reported anxiety (EPDS-3A ≥7)	2.1 (140)	7.8 (8; 7–8)

^a^ Assessed at 3 months postpartum

^b^ Self-identified anxiety at least once at 10 days, 1 month *or* 3 months post-partum

^c^ Self-identified anxiety at 10 days, 1 month *and* 3 months post-partum

### Agreement between self-identified anxiety and self-reported anxiety

**Fig. 2:**
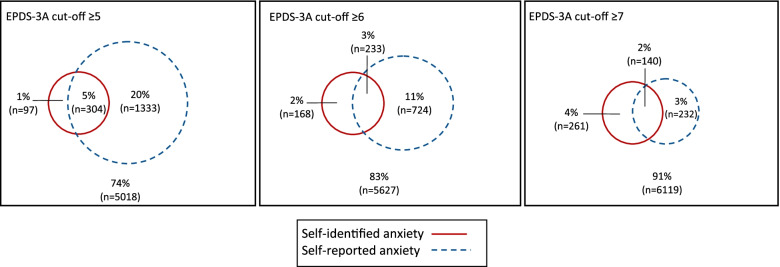
Overlap between self-identified anxiety and self-reported anxiety at different cut-offs of the EPDS anxiety subscale (EPDS-3A)

Table 2 summarises the agreement between self-identified and self-reported anxiety. Among those with self-identified anxiety at 3 months postpartum, 58.1% had EPDS-3A scores ≥6. Of those with EPDS-3A scores ≥6, 24.4% had self-identified anxiety. Statistical agreement between the two measures was minimal with Cohen’s kappa coefficients of 0.224. 0.283 and 0.324 for cut-offs of ≥5, ≥6 and ≥7, respectively. Figure 2 shows the changing overlap between measures with increasing EPDS thresholds.

**Table 2:** Agreement between postnatal self-identified anxiety and self-reported anxiety at different cut-offs of the EPDS anxiety subscale (EPDS-3A)

	Proportion with self-identified anxiety	Proportion with self-reported anxiety
**Self-identified anxiety**	–	EPDS-3A ≥5: 75.8% (304/401)
		EPDS-3A ≥6: 58.1% (233/401)
		EPDS-3A ≥7: 34.9% (140/401)
**Self-reported anxiety**	EPDS-3A≥5: 18.6% (304/1637)	–
	EPDS-3A≥6: 24.4% (233/957)	
	EPDS-3A≥7: 37.6% (140/372)

### Factors associated with self-identified anxiety and self-reported anxiety

Table 3 presents the socio-demographic, obstetric and psychological characteristics of women with self-identified anxiety and those with self-reported anxiety. In the multivariable logistic regression analysis, the following variables were statistically significantly associated with both self-identified and self-reported anxiety: postpartum PTSD-type symptoms (adjusted odds ratio [aOR] 5.12 in self-identified; 2.14 in self-reported); antenatal anxiety (aOR 3.11 in self-identified; 2.24 in self-reported); postpartum depression (aOR 2.23 in self-identified; 2.54 in self-reported); fatigue (aOR 1.99 in self-identified; 1.71 in self-reported); and sleep problems (sleep problems (aOR 1.93 in self-identified; 1.78 in self-reported). The following variables were significantly associated only with self-identified anxiety: physical problems (aOR 1.84); being of Black or minority ethnicity (BME) background (aOR 0.39). The following factors were associated only with self-reported anxiety: being a Northern Ireland survey respondent (compared with England respondent; aOR 1.81); having a mixed or unhappy reaction to the pregnancy (aOR 1.65); living without a partner (aOR 1.37); and antenatal depression (aOR 1.32). Among both self-identified and self-reported anxiety groups, psychological factors were the strongest associated factors.

**Table 3:** Socio-demographic, obstetric and psychological characteristics of women with and without self-identified anxiety and self-reported anxiety (EPDS-3A scores ≥6)

	Self-identified anxiety				Self-reported anxiety (EPDS-3A score ≥6)			
	Anxiety (*N* = 401)^a^ n (%)	No anxiety (*N* = 6351) n (%)	uOR (95% CI)	aOR (95% CI)	EPDS-3A ≥ 6 (*N* = 957) n (%)	EPDS-3A < 6 (*N* = 5795) n (%)	uOR (95% CI)	aOR (95% CI)^b^
**Socio-demographic factors**								
**Age**				–				–
< 20 years	4 (3.4)	114 (96.6)	0.54 (0.20–1.49)		24 (20.3)	94 (79.7)	**1.69 (1.07–2.67)**	
20–29 years	131 (5.7)	2177 (94.3)	0.93 (0.75–1.16)		368 (15.9)	1940 (84.1)	**1.26 (1.09–1.45)**	
30–39 years	238 (6.1)	3689 (93.9)	Ref		515 (13.1)	3412 (86.9)	Ref	
≥ 40 years	28 (7.0)	371 (93.0)	1.17 (0.78–1.76)		50 (12.5)	349 (87.5)	0.95 (0.70–1.29)	
**Education**				–				–
≤ 16 years	47 (5.1)	882 (94.9)	0.82 (0.60–1.12)		150 (16.2)	779 (83.8)	1.20 (0.99–1.45)	
≥ 17 years	353 (6.1)	5421 (93.9)	Ref		800 (13.9)	4974 (86.1)	Ref	
**Ethnicity**								–
White British	376 (6.3)	5616 (93.7)	Ref	Ref	840 (14.0)	5152 (86.0)	Ref	
BME	22 (3.5)	614 (96.5)	**0.54 (0.35–0.83)**	**0.39 (0.24–0.61)**	100 (15.7)	536 (84.3)	1.14 (0.91–1.43)	
**Birth country**				–				–
UK	341 (6.3)	5043 (93.7)	Ref		769 (14.3)	4615 (85.7)	Ref	
Outside UK	52 (4.3)	1159 (95.7)	**0.66 (0.49–0.89)**		168 (13.9)	1043 (86.1)	0.97 (0.81–1.16)	
**Help with English**^c^				–				–
Does not need help	394 (6.0)	6124 (94.0)	Ref		923 (14.2)	5595 (85.8)	Ref	
Needs help	2 (1.1)	173 (98.9)	**0.18 (0.04–0.73)**		25 (14.3)	150 (85.7)	1.01 (0.66–0.55)	
**Living with partner**				–				
With partner	354 (6.0)	5561 (94.0)	Ref		777 (13.1)	5138 (86.9)	Ref	Ref
Without partner	47 (5.6)	790 (94.4)	0.93 (0.68–1.28)		180 (21.5)	657 (78.5)	**1.81 (1.51–2.17)**	**1.37 (1.11–1.69)**
**Survey country**				–				
England	246 (5.9)	3932 (94.1)	Ref		557 (13.3)	3621 (86.7)	Ref	Ref
Northern Ireland	155 (6.0)	2419 (94.0)	1.02 (0.83–1.26)		400 (15.5)	2174 (84.5)	**1.20 (1.04–1.37)**	**1.81 (1.52–2.15)**
**Obstetric factors**								
**Parity**				–				–
Primiparous	165 (6.3)	2440 (93.7)	Ref		380 (14.6)	2225 (85.4)	Ref	
Multiparous	233 (5.8)	3794 (94.2)	0.91 (0.74–1.12)		555 (13.8)	3472 (86.2)	0.94 (0.81–1.08)	
**Planned pregnancy**				–				**–**
Planned	306 (5.8)	4936 (94.2)	Ref		288 (19.7)	1173 (80.3)	Ref	
Unplanned	94 (6.4)	1367 (93.6)	1.11 (0.87–1.41)		661 (12..6)	4581 (87.4)	**0.70 (1.46–1.98)**	
**Reaction to pregnancy**				–				
Happy	306 (5.5)	5279 (94.5)	Ref		680 (12.2)	4905 (87.8)	Ref	Ref
Mixed or unhappy	91 (8.2)	1025 (91.9)	**1.53 (1.20–1.95)**		261 (23.4)	855 (76.6)	**2.20 (1.88–2.58)**	**1.65 (1.37–1.97)**
**Birth as expected**				–				–
As expected	99 (4.8)	1948 (95.2)	Ref		254 (12.4)	1793 (87.6)	Ref	
Better than expected	144 (5.1)	2695 (94.9)	**1.05 (1.49–2.52)**		364 (12.8)	2475 (87.2)	1.04 (0.87–1.23)	
Worse than expected	148 (9.0)	1506 (91.0)	1.93 (0.81–1.37)		310 (18.7)	1344 (81.3)	**1.63 (1.36–1.95)**	
**Chronic condition in pregnancy**				–				**–**
No	339 (5.6)	5770 (94.4)	Ref		837 (13.7)	5272 (86.3)	Ref	
Yes	58 (10.1)	516 (89.9)	**1.91 (1.43–2.56)**		111 (19.3)	463 (80.7)	**1.51 (1.21–1.88)**	
**Pregnancy complications**				–				**–**
No	265 (5.5)	4562 (94.5)	Ref		657 (13.6)	4170 (86.4)	Ref	
Yes	126 (7.1)	1661 (93.0)	**1.31 (1.05–1.63)**		283 (15.8)	1504 (84.2)	**1.19 (1.03–1.39)**	
**DeliveryBirth type**				–				–
Normal vaginal	201(5.3)	3599 (94.7)	Ref		495 (13.0)	3305 (87.0)	Ref	
Instrumental	64 (6.3)	952 (93.7)	1.20 (0.90–1.61)		157 (15.5)	859 (84.5)	**1.22 (1.00–1.48)**	
Planned CS	61 (6.9)	822 (93.1)	1.33 (0.99–1.79)		129 (14.6)	754 (85.4)	1.14 (0.93–1.41)	
Emergency CS	387 (7.5)	758 (92.5)	**1.44 (1.07–1.94)**		139 (17.0)	680 (83.0)	**1.36 (1.11–1.68)**	
**NICU admission**				–				**–**
No	319 (5.8)	5191 (94.2)	Ref		768 (13.9)	4742 (86.1)	Ref	
Yes	55 (8.2)	620 (91.9)	**1.44 (1.07–1.94)**		116 (17.2)	559 (82.8)	**1.28 (1.03–1.59)**	
**Physical problems PP**								**–**
No	22 (2.0)	1095 (98.0)	Ref	Ref	91 (8.2)	1026 (91.8)	Ref	
Yes	379 (6.7)	5256 (93.3)	**3.59 (2.32–5.54)**	**1.84 (1.14–2.96)**	866 (15.4)	4769 (84.6)	**2.05 (1.63–2.57)**	
**Psychological factors**								
**Antenatal depression**				–				
No	333 (5.3)	5990 (94.7)	Ref		800 (12.7)	5523 (87.4)	Ref	Ref
Yes	68 (15.9)	361 (84.1)	**3.39 (2.56–4.49)**		127 (36.6)	272 (63.4)	**3.98 (3.23–4.92)**	**1.32 (1.01–1.73)**
**Antenatal anxiety**								
No	240 (4.1)	5654 (95.9)	Ref	**Ref**	660 (11.2)	5234 (88.8)	Ref	Ref
Yes	161 (18.8)	697 (81.2)	**5.44 (4.39–6.74)**	3.11 (2.45-3.94)	297 (34.6)	561 (65.4)	**4.20 (3.57–4.94)**	**2.24 (1.83–2.75)**
**Fatigue 3 m PP**								
No	3095 (97.6)	77 (2.4)	Ref	**Ref**	247 (7.8)	2925 (92.2)	Ref	Ref
Yes	3256 (90.9)	324 (9.1)	**4.00 (3.10–5.15)**	(1.99 (1.50-2.63)	710 (19.8)	2870 (80.2)	**2.93 (2.51–3.42)**	**1.71 (1.45–2.03)**
**Depression 3 m PP**								
No	153 (3.2)	4599 (96.8)	Ref	**Ref**	446 (9.4)	4306 (90.6)	Ref	Ref
Yes	248 (12.4)	1752 (87.6)	**4.25 (3.45–5.24)**	2.23 (1.76-2.81)	511 (25.6)	1489 (74.4)	**3.31 (2.88–3.81)**	**2.54 (2.13–3.04)**
**Sleep problems 3 m PP**								
No	277 (4.6)	5763 (95.4)	Ref	**Ref**	712 (11.8)	5328 (88.2)	Ref	Ref
Yes	124 (17.4)	588 (82.6)	**4.39 (3.49–5.51)**	1.93 (1.48-2.50)	245 (34.4)	467 (65.6)	**3.93 (3.30–4.67)**	**1.78 (1.45–2.17)**
**PTSD symptoms 3 m PP**								
No	262 (4.4)	5755 (95.6)	Ref	**Ref**	690 (11.5)	5327 (88.5)	Ref	Ref
Yes	139 (18.9)	596 (81.1)	**5.12 (4.10–6.40)**	2.25 (1.75-2.90)	267 (36.3)	468 (63.7)	**4.40 (3.72–5.22)**	**2.14 (1.76–2.60)**

Bold denotes statistical significance at p < 0.10 for univariable analysis (uOR) and p < 0.05 for multivariable analysis (aOR)

*Abbreviations*: *aOR* adjusted odds ratio, *BME* Black or minority ethnic, *CS* Caesarean section, *EPDS-3A* Edinburgh Postnatal Depression Scale (anxiety subscale), *m* month, *NICU* neonatal intensive care unit, *PP* postpartum, *PTSD* Post-Traumatic Stress Disorder, *uOR* unadjusted odds ratio

^a^ Number who responses to each item varies; some variables may not add up to total denominator

^b^ Adjusted odds ratio shown for variables statistically significantly (*p* < 0.10) associated with the outcome in univariable analysis

^c^ Not included in multivariable regression model doe self-reported anxiety due to small numbers

## Discussion

Our findings highlight important differences between two approaches to identify women with anxiety. We compared the use of a single, direct question asking women whether they self-identify as having anxiety against  a self-report measure of anxiety using the EPDS-3A. In our sample of postnatal women in England and Northern Ireland, at 3 months postpartum 5.9% of women had self-identified anxiety, 14.2% had self-reported anxiety using an EPDS-3A cut-off of ≥6, and 3.5% were positive on both measures. Approximately half (58.1%) of those with self-identified anxiety also had self-reported anxiety, while a quarter (24.4%) of those with self-reported anxiety also had self-identified anxiety. Despite this overlap, the overall statistical agreement was minimal suggesting that the two approaches to assessing anxiety identify different groups of women.

The prevalence of self-reported anxiety of 14.2% at 3 months postpartum is in line with previous estimates [2, 3]. The declining proportion of women with self-identified anxiety during the first 3 months postpartum is encouraging and follows the trend in self-identified postnatal depression reported in an earlier analysis [9]. More concerning are the low levels of self-identified anxiety and the minimal agreement between the two measures. We found similarly low levels of agreement in our previous analysis of depression measures in this sample, in which 6.1% had self-identified depression, 9.1% had self-reported depression and only 2.8% were positive on both measures [9]. Low rates of self-identified anxiety in our current analysis could suggest low awareness of the symptoms which constitute anxiety. In the postnatal period, feelings of worry and uncertainty are common and women may blame themselves during difficult periods. Women may experience symptoms of anxiety – which are captured by the EPDS-3A – but not recognising them as unusual and not attributing them to anxiety. Understanding of perinatal anxiety is variable even among healthcare professionals, and programmes of education – for midwives, obstetricians or general practitioners, for example – may have a limited or inconsistent inclusion of perinatal mental health issues [6]. There also remains uncertainty around what constitutes ‘risk’ and the need for additional care in pregnancy. Women experiencing anxiety may also under-report this on the self-identification measure due to fear of being judged or worry that this would be considered a sign of poor parenting [24, 25]. Answering ‘yes’ to the individual symptoms of anxiety as elicited by the EPDS – which women may or may not associate with anxiety – may be perceived as more acceptable than self-identifying as having anxiety. This was reported by a qualitative study of perinatal women in the UK which found that some women welcomed the less personal means of disclosure offered by screening tools [26].

Conversely, the discrepancy in prevalence of self-identified and self-reported anxiety may be a result of the EPDS-3A over-estimating the number of women with significant anxiety. For some women, anxiety symptoms are brief and short-lived and the EPDS-3A may be capturing symptoms which in fact are only transient and not indicative of an anxiety disorder. The binary nature of cut-offs on self-report measures such as the EPDS means that care must be taken in establishing the appropriate cut-off. Our exploratory analyses illustrate how decreasing the EPDS-3A threshold resulted in a greater overlap between women with raised EPDS scores and those who self-identified as anxious. As with all screening instruments, the optimal threshold of the EPDS-3A varies according to culture, geographical contexts and participant characteristics [27]. In a study of antenatal women in Australia, for example, Swalm et al. (2010) found an EPDS-3A cut-off of ≥4 best captured the top quartile of their population [18]. However, lowering the threshold risks identifying women with subclinical anxiety who do not need the same level of clinical follow-up or support as those with higher scores. This is not always appropriate and risks overwhelming mental health services. Rather, women with lower scores might benefit from a different type of support intervention and preventative education for future pregnancies. While we have focused on the identification of women with anxiety in clinical settings, it is important to note that standardised self-report measures have a significant role to play in research on maternal mental health, especially in population-based studies that contribute to identifying needs and maternity care provision planning more broadly.

Psychosocial factors including antenatal depression, antenatal anxiety, postnatal fatigue, sleep problems and PTSD-type symptoms were strongly associated with both self-identified and self-reported anxiety. These associations are unsurprising and suggest that women with anxiety experience a cluster of symptoms. Overlap between anxiety and depressive symptoms is common and co-morbidity between the two disorders is high; similarly, overlap between anxiety and PTSD-type symptoms is also high [28, 29]. The higher levels of self-reported anxiety among Northern Irish women may reflect a higher prevalence of mental disorders among the general population: a Northern Ireland Health Survey conducted in 2014–2015 reported a 25% higher prevalence of mental health disorders than England, with higher prevalence among women than men [30]. Women from a BME background were less than half as likely as White British women to self-identify as having anxiety. This is concerning given that across many settings, higher prevalence of perinatal mental disorders are seen among marginalised and minority groups [31, 32]. The small number of BME mothers in our sample means that this finding must be interpreted with caution, but nevertheless the low levels of self-identified anxiety combined with EPDS-3A scores similar to non-BME women may indicate that BME groups are particularly unlikely to attribute anxiety symptoms to anxiety. There may be less information available among BME groups around what constitutes anxiety or greater levels of fear and perceived stigma over reporting anxiety [32, 33]. Our findings suggest that BME women experiencing anxiety might be more likely to be identified through a self-report measure such as the EPDS, which is less obviously about anxiety, rather than through a direct question about anxiety. Care must be taken, however, to ensure that self-report measures used are culturally-sensitive, appropriate and valid [33].

Women who score above the EPDS-3A cut-off but do not self-identify as having anxiety may be experiencing clinically significant anxiety, even though they may not recognise their symptoms as such. These women require further assessment which could be in the form of a clinical interview or using a tool such as the Generalised Anxiety Disorder (GAD-7) scale, the latter ideally being administered at two separate time points [34]. Women who self-identify as having anxiety but score below the EPDS threshold are also likely to benefit from some form of follow-up and support. These women may be feeling overwhelmed during what can be a challenging transition period. Creating an opportunity for health professionals to verbally ask women directly whether they feel anxious may enable more women with anxiety to be identified. There may be further benefits to such a direct approach; for example, it may provide an opportunity for the health professional to ask follow-up questions, understand any underlying contributors to the anxiety, respond empathetically and give appropriate advice and support. This could be especially important in settings where uptake of referral to formal mental health support is low. What women think about their mental health status matters; regardless of their scores on screening instruments, women who feel they are experiencing anxiety should have the opportunity to further discuss their symptoms and needs. Follow-up might be by a health professional or through repeat screening at a second timepoint. While for some women formal counselling or mental health support might be appropriate, for others referral to non-clinical, self-help or peer support interventions may be more suitable. Finally, the antenatal period also offers an opportunity to raise issues around perinatal mental health during antenatal education sessions [35]. Ensuring that women have family and community support in place to help them navigate this transition time is important and will help confirm that their concerns, worries and symptoms are recognised, validated and addressed.

### Strengths and limitations

This paper’s strength is the population-based design which enables the experiences of a wide range of women to be captured. A limitation is the survey response rate of 47% in England and 45% in Northern Ireland, and the under-representation of women from marginalised groups, those living in poverty and those born outside of the UK [10, 11]. Previous research has highlighted these groups as being at higher risk of poor perinatal mental health and our analyses may have missed important associations. The data was collected in 2014, and prevalence and associations identified in our analyses may be different from current trends. Although we define our outcome as anxiety at 3 months post-partum, the questionnaire reminders required for some women and the lag in time between women receiving and completing their questionnaires means that women may have been between three and 6 months post-partum when responding. Finally, we were unable to explore the accuracy of either anxiety measure as there was no diagnostic interview against which to compare them. However, for the current analysis we were interested in comparing a self-report measure with a direct question and it was not within the remit of our research question to assess either of these measures against a clinical diagnosis.

## Conclusion

Identifying women who may be experiencing anxiety during the perinatal period is important in order to offer appropriate and timely support. Our comparison of self-identified and self-reported anxiety suggests that despite some overlap, these two approaches identify different groups of women. Low levels of self-identified anxiety may suggest low awareness of the symptoms that constitute anxiety, particularly in the perinatal period. This highlights an important opportunity to discuss and address perinatal mental health in the antenatal period. Women who score above the cut-off on self-report measures require further assessment, while those who self-identify as having anxiety may also benefit from follow-up and other forms of support. Further evidence comparing the two assessment measures to a clinical diagnosis would help to increase our understanding of these different groups of women and their respective needs for support.

## Data Availability

The datasets generated and/or analysed during the current study are not publicly available due to the scope of the consent obtained from study participants restricting our ability to share the data on ethical and legal grounds but NMS data are available from the corresponding author on reasonable request.

## Abbreviations

BME: Black and minority ethnic
EPDS: Edinburgh Postnatal Depression Scale
EPDS-3A: Edinburgh Postnatal Depression Scale anxiety subscale
GAD-7: Generalized Anxiety Disorder scale
NICE: National Institute of Health and Clinical Excellence
NMS: National Maternity Survey
PTSD: Post-traumatic stress disorder

## References

[1] Harrison S, Alderdice F (2020). Challenges of defining and measuring perinatal anxiety. J Reprod Infant Psychol.

[2] Dennis CL, Falah-Hassani K, Shiri R (2017). Prevalence of antenatal and postnatal anxiety: systematic review and meta-analysis. Br J Psychiatry.

[3] Fawcett EJ, Fairbrother N, Cox ML, White IR, Fawcett JM. The prevalence of anxiety disorders during pregnancy and the postpartum period: a multivariate Bayesian meta-analysis. J Clin Psychiatry. 2019;80(4):18r12527. 10.4088/JCP.18r12527. 10.4088/JCP.18r12527 PMC6839961 31347796

[4] Aboraya A, Nasrallah H, Muvvala S, El-Missiry A, Mansour H, Hill C (2016). The standard for clinicians’ interview in psychiatry (SCIP): a clinician-administered tool with categorical, dimensional, and numeric output-conceptual development, design, and description of the SCIP. Innov Clin Neurosci.

[5] NICE (2014). (National Institute for health and care excellence). Antenatal and postnatal mental health: clinical management and service guidance (CG192).

[6] Silverwood V, Nash A, Chew-Graham CA, Walsh-House J, Sumathipala A, Bartlam B (2019). Healthcare professionals’ perspectives on identifying and managing perinatal anxiety: a qualitative study. Br J Gen Pract.

[7] Bemme D, D'Souza NA (2014). Global mental health and its discontents: an inquiry into the making of global and local scale. Transcult Psychiatry.

[8] Ali GC, Ryan G, De Silva MJ (2016). Validated screening tools for common mental disorders in low and middle income countries: a systematic review. PLoS One.

[9] Fellmeth G, Opondo C, Henderson J, Redshaw M, McNeill J, Lynn F, et al. Identifying postnatal depression: comparison of a self-reported depression item with Edinburgh Postnatal Depression Scale scores at three months postpartum. J Affect Disord. 2019;251:8–14. 10.1016/j.jad.2019.03.002 30889476

[10] Alderdice F, Hamilton K, McNeill J, Lynn F, Curran R, Redshaw M. Birth NI: a survey of women’s experience of maternity care in Northern Ireland.: School of Nursing and Midwifery. Belfast: Queen’s University Belfast; 2016. Available at:http://www.qub.ac.uk/schools/SchoolofNursingandMidwifery/FileStore/Filetoupload,670193,en.pdf?platform=hootsuite.

[11] Redshaw M, Henderson J. Safely delivered: a national survey of women’s experience of maternity care 2014. Oxford: National Perinatal Epidemiology Unit; 2015. Available at: https://www.npeu.ox.ac.uk/downloads/files/reports/Safely%20delivered%20NMS%202014.pdf.

[12] Dillman DA (2007). Mail and internet surveys. The tailored design method.

[13] Garcia J, Redshaw M, Fitzsimmons B. First class delivery : a national survey of women's views of maternity care. London: Audit Commission; 1998.

[14] Redshaw M, Rowe R, Hockley C, Brocklehurst P (2007). Recorded delivery: a national survey of women's experience of maternity care, 2006.

[15] Commission H (2008). Towards better births: a review of maternity services in England.

[16] Cox JL, Holden JM, Sagovsky R (1987). Detection of postnatal depression. Development of the 10-item Edinburgh Postnatal Depression Scale. Br J Psychiatry.

[17] Bowen A, Bowen R, Maslany G, Muhajarine N (2008). Anxiety in a socially high-risk sample of pregnant women in Canada. Can J Psychiatr.

[18] Swalm D, Brooks J, Doherty D, Nathan E, Jacques A (2010). Using the Edinburgh postnatal depression scale to screen for perinatal anxiety. Arch Womens Ment Health.

[19] Matthey S, Fisher J, Rowe H (2013). Using the Edinburgh postnatal depression scale to screen for anxiety disorders: conceptual and methodological considerations. J Affect Disord.

[20] Martin CR, Redshaw M (2018). Establishing a coherent and replicable measurement model of the Edinburgh postnatal depression scale. Psychiatry Res.

[21] Matthey S (2008). Using the Edinburgh postnatal depression scale to screen for anxiety disorders. Depress Anxiety.

[22] Henderson J, Redshaw M (2013). Anxiety in the perinatal period: antenatal and postnatal influences and women’s experience of care. J Reprod Infant Psychol.

[23] McHugh ML (2012). Interrater reliability: the kappa statistic. Biochem Med (Zagreb).

[24] Oh S, Chew-Graham CA, Silverwood V, Shaheen SA, Walsh-House J, Sumathipala A (2020). Exploring women's experiences of identifying, negotiating and managing perinatal anxiety: a qualitative study. BMJ Open.

[25] Staneva AA, Wigginton B (2018). The happiness imperative: exploring how women narrate depression and anxiety during pregnancy. Fem Psychol.

[26] Button S, Thornton A, Lee S, Shakespeare J, Ayers S (2017). Seeking help for perinatal psychological distress: a meta-synthesis of women's experiences. Br J Gen Pract.

[27] Gibson J, McKenzie-McHarg K, Shakespeare J, Price J, Gray R (2009). A systematic review of studies validating the Edinburgh postnatal depression scale in antepartum and postpartum women. Acta Psychiatr Scand.

[28] O'Hara MW, Wisner KL (2014). Perinatal mental illness: definition, description and aetiology. Best Pract Res Clin Obstet Gynaecol.

[29] Agius A, Xuereb RB, Carrick-Sen D, Sultana R, Rankin J (2016). The co-existence of depression, anxiety and post-traumatic stress symptoms in the perinatal period: a systematic review. Midwifery..

[30] Mental Health Foundation (2016). Mental health in Northern Ireland: fundamental facts 2016.

[31] Gennaro S, O’Connor C, McKay EA, Gibeau A, Aviles M, Hoying J (2020). Perinatal anxiety and depression in minority women. MCN Am J Matern Child Nurs.

[32] Watson H, Harrop D, Walton E, Young A, Soltani H (2019). A systematic review of ethnic minority women's experiences of perinatal mental health conditions and services in Europe. PLoS One.

[33] Memon A, Taylor K, Mohebati LM, Sundin J, Cooper M, Scanlon T (2016). Perceived barriers to accessing mental health services among black and minority ethnic (BME) communities: a qualitative study in Southeast England. BMJ Open.

[34] Spitzer RL, Kroenke K, Williams JB, Löwe B (2006). A brief measure for assessing generalized anxiety disorder: the GAD-7. Arch Intern Med.

[35] Grussu P, Quatraro RM, Jorizzo GJ (2020). Supporting perinatal women in the context of the COVID-19 emergency: can web-based antenatal education classes make it possible?. J Reprod Infant Psychol..

